# Mapping an intelligent algorithm for predicting female adolescents’ cervical vertebrae maturation stage with high recall and accuracy

**DOI:** 10.1186/s40510-024-00523-5

**Published:** 2024-05-21

**Authors:** Huayu Ye, Hongrui Qin, Ying Tang, Nicha Ungvijanpunya, Yongchao Gou

**Affiliations:** 1 Department of Orthodontics, Stomatological Hospital of Chongqing Medical University, 426#, Songshi North Road, Yubei District, Chongqing, 401147 P.R. China; 2 Chongqing Key Laboratory of Oral Diseases and Biomedical Sciences, 426#, Songshi North Road, Yubei District, Chongqing, 401147 P.R. China; 3 Chongqing Municipal Key Laboratory of Oral Biomedical Engineering of Higher Education, 426#, Songshi North Road, Yubei District, Chongqing, 401147 P.R. China; 4 Faculty of Dentistry, Chulalongkorn University, 34 Henri Dunant Road, Pathumwan, Bangkok, 10330 Thailand

**Keywords:** CVM method, Skeletal maturation, Cervical vertebrae, Lateral cephalogram

## Abstract

**Backgrounds and objectives:**

The present study was designed to define a novel algorithm capable of predicting female adolescents’ cervical vertebrae maturation stage with high recall and accuracy.

**Methods:**

A total of 560 female cephalograms were collected, and cephalograms with unclear vertebral shapes and deformed scales were removed. 480 films from female adolescents (mean age: 11.5 years; age range: 6–19 years) were used for the model development phase, and 80 subjects were randomly and stratified allocated to the validation cohort to further assess the model’s performance. Derived significant predictive parameters from 15 anatomic points and 25 quantitative parameters of the second to fourth cervical vertebrae (C2-C4) to establish the ordinary logistic regression model. Evaluation metrics including precision, recall, and F1 score are employed to assess the efficacy of the models in each identified cervical vertebrae maturation stage (iCS). In cases of confusion and mispredictions, the model underwent modification to improve consistency.

**Results:**

Four significant parameters, including chronological age, the ratio of D3 to AH3 (D3:AH3), anterosuperior angle of C4 (@4), and distance between C3lp and C4up (C3lp-C4up) were administered into the ordinary regression model. The primary predicting model that implements the novel algorithm was built and the performance evaluation with all stages of 93.96% for accuracy, 93.98% for precision, 93.98% for recall, and 93.95% for F1-score were obtained. Despite the hybrid logistic-based model achieving high accuracy, the unsatisfactory performance of stage estimation was noticed for iCS3 in the primary cohort (89.17%) and validation cohort (85.00%). Through bivariate logistic regression analysis, the posterior height of C4 (PH4) was further selected in the iCS3 to establish a corrected model, thus the evaluation metrics were upgraded to 95.83% and 90.00%, respectively.

**Conclusions:**

An unbiased and objective assessment of the cervical vertebrae maturation (CVM) method can function as a decision-support tool, assisting in the evaluation of the optimal timing for treatment in growing adults. Our novel proposed logistic model yielded individual formulas for each specific CVM stage and attained exceptional performance, indicating the capability to function as a benchmark for maturity evaluation in clinical craniofacial orthopedics for Chinese female adolescents.

## Background

Each individual has a unique growth pattern, which is constituted of certain periods of growth accelerations and decelerations. The onset and duration of the mentioned period vary between skeletal classes, adding complexity to the orthodontic intervention. By initiating treatment at the patient’s optimal skeletal maturational stage, a favorable outcome with minimum risk of unwanted effects is expected [1]. The pubertal growth spurt can be determined by chronological age and secondary sexual maturation as well as mandibular growth, weight and height, menarche and voice changes, and cervical vertebral maturation (CVM) [2–5].

The CVM method, based on the morphology of three cervical vertebrae, is a common approach to predicting the timing of pubertal growth, as well as estimating growth velocity and the proportion of growth [6, 7]. Early in 1972, Lamparski published an atlas to display changes in cervical vertebrae for evaluating individual skeletal maturation and concluded that the CVM method is a valid indicator for the assessment of skeletal maturity and is comparable with hand-wrist radiographs, with the additional benefit of avoiding additional radiation exposure [8]. Baccetti et al. further implemented a modification of the CVM method to make it clearer, easier, and more applicable to the majority of patients, that is, a more limited number of vertebral bodies and more accurate definitions of stages to avoid the comparative assessment of between-stage variations [9]. The newly proposed CVM method consisted of six maturational stages with comparable high reliability and validity in the clinical assessment of skeletal maturity. Nevertheless, the available literature regarding the reproducibility of this staging method is controversial, with intra-observer and inter-observer correlations ranging from perfect to poor agreement [10, 11]. Reasons for the poor reliability have been attributed to the level of training, clinician experience, and evaluation metrics based on simple qualitative analysis of the vertebral shape and size.

In the pursuit of precise and objective predictions, several mathematical models were developed, as manual assessment of the shape of cervical vertebrae has been criticized for its tediousness and variabilities. Previous proposed regression-based attempts were based on the linear form [12, 13]; however, the methodology was limited to a collection with most of the CVM features, lacking the capability to precisely detect the exact maturational stage. Santiago et al. highlighted that the methodological deficiencies in the analysis of skeletal maturation did not involve any form of randomization, blinding, or sample size calculation [14]. The previously proposed formulae are demonstrated to vary with polymorphism and sexual dimorphism and display several algorithmic errors, which limit their clinical predictive use of the CVM staging method [15, 16]. Recent advances have seen intelligent machine learning algorithms emerge to assist in medical diagnosis, thus selecting the right technique is crucial, considering the anticipated outcome or prediction [17]. The aim of the present study, therefore, is to establish and validate a formula in adolescent female groups to facilitate the applicability of the CVM method to determine the skeletal age. Furthermore, correlation analysis, widely employed in previous studies, is not sufficient to reliably assess the diagnostic performance for the identification of the maturation phase in individual subjects [18]. Thus, a dedicated analysis of model performance is needed, which encompasses adequate accuracy, reproducibility, and correlation analysis, as well as appropriate sensitivity-specificity analysis [19]. The CVM method we utilized is based on Baccetti’s method [20, 21] and comprised of four maturational stages (iCS1 through iCS4, instead of Stage 1 through Stage 6 in the former CVM method), with the peak in mandibular growth occurring between iCS2 and iCS3. Thus, the assessment of the CVM stage through the comparative analysis of between-stage changes should be avoided, which improves the accuracy and repeatability. In our context, we evaluated the proposed estimation model of the CVM stage grounded on the standard with accuracy and confusion matrix analysis, thus indicating the appropriate and ideal stages for clinical interventions.

## Materials and methods

### Study design and patient selection

**Fig. 1:**
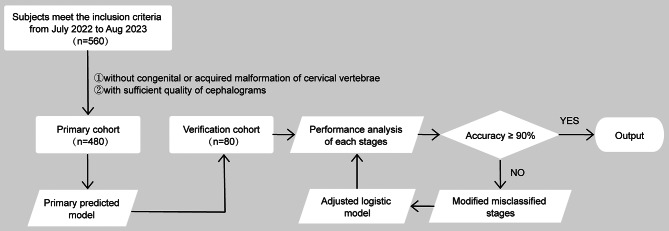
Overview of the progress in primary model development for CVM stage estimation. 480 films from female adolescents were randomly selected for the model development phase, and 80 subjects were then allocated to the validation cohort to further evaluate the model’s performance. After evaluating the performance of primary models corresponding to each stage, we identified misclassified stages when the observed percentage fell below our decision threshold (90.00%)

The present retrospective study was designed to derive an algorithm to determine cervical vertebral bone age for female adolescents of ethics in the southern and southeast parts of China. The input data for developing algorithms were chronological age and the morphological dimensions of the cervical vertebrae from lateral cephalograms. The output was the accuracy of the logistic model for predicting the maturation stages. The flow diagram of patient selection and study design is shown in Fig. 1. All procedures performed were following the ethical standards of the Clinical Research Ethics Committee of Chongqing Medical University (Approval No.2022-070); consent waived as there was no intervention; data analyzed in an anonymous form. A total of 560 sets of existing pretreatment cephalograms taken from July 2022 to Aug 2023 were recruited (mean age: 11.5 years; age range: 6–19 years). Patients with no congenital or acquired malformation of the cervical vertebrae and cephalograms of sufficient quality were included. Those with evidence of gross skeletal asymmetries or bone disease were excluded from the study. Patients were seated and positioned in the natural head posture during imaging. Cephalograms were obtained from patients using the KODAK DirectView DR9000 with standard radiographic exposure procedures, employing specific parameters: 80 kV voltage, 10 mA current, and 0.5 s exposure time. All personal information, except age and sex, was removed from the data.

### Skeletal maturation assessment and data acquisition

Skeletal maturation entails alterations in the size of vertebral bodies and the shape of the upper and lower borders of C2, C3, and C4 vertebrae. These changes have been categorized into six stages, which correlate with morphological modifications of the vertebral shapes and estimated time lapse from the mandibular growth peak. To streamline and enhance the applicability of our newly proposed method, each stage was easily identified in the process of model development.

iCS1 = before mandibular growth peak.

iCS2 = during the year of the mandibular growth peak.

iCS3 = 1 or 2 years after mandibular growth peak.

iCS4 = after the end of mandibular growth peak.

**Fig. 2:**
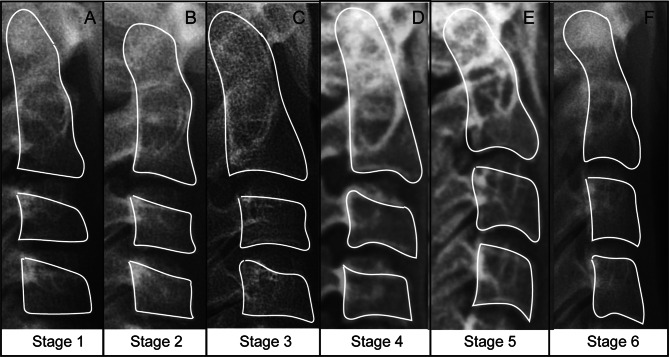
Cervical vertebral maturation staging system. (**A**) Stage 1 (initiation): vertebrae are wedge-shaped and inferior borders of bodies of the second (C2), third (C3), and fourth (C4) cervical vertebrae are flat; (**B**) Stage 2 (acceleration): concavities develop on the inferior borders of C2 and C3; bodies of C3 and C4 are nearly rectangular, but the inferior border of C4 is still flat; (**C**) Stage 3 (transition): clear concavities develop on inferior borders of C2 and C3; (**D**) Stage 4 (deceleration): distinct concavities are seen on inferior borders of C2, C3, and C4 and bodies of all cervical vertebrae are rectangular; (**E**) Stage 5 (maturation): vertebral bodies of C3 and C4 are nearly square and spaces between vertebral bodies are reduced. (**F**) Stage 6 (completion): vertebral bodies of C3 and C4 are more vertical than horizontal

Briefly, stages 1–2 were classified as iCS 1 (prepuberty); stage 3 was classified as iCS 2 (pubertal peak); stage 4 was classified as iCS 3 (pubertal slowdown); stages 5–6 were classified as iCS 4 (post-puberty). In the event of misclassification, where the features of two consecutive stages were present in a single image, the intermediate or in-between stages were included in the more mature stage. Two experienced orthodontists with 5 years of clinical practice simultaneously evaluated the CVM stage for each cephalogram strictly following the descriptions of Baccetti [9]. The ground-truth iCS stage was determined as frequently chosen by the experts. Any disagreement was resolved by inter-examiner discussion or consulting a third orthodontist with 20 years of experience (detailed in Table 1; Fig. 2).

**Table 1:** Cervical vertebral morphologic features of each maturation stage and classification in our proposed method

CVM stage	Inferior border’ shape				Morphology		Clinical implication	Classification	
	C2	C3		C4	C3	C4			
Stage 1	F	F	F		T	T	Prepuberty		iCS 1
Stage 2	C	F	F		T	T			iCS 1
Stage 3	C	C	F		T	T, RH	Pubertal peak		iCS 2
Stage 4	C	C	C		RH	RH	Pubertal slowdown		iCS 3
Stage 5	C	C	C		RH, S	RH, S	Post-puberty		iCS 4 iCS 4
Stage 6	C	C	C		RH, RV	RH, RV			

C: concavity; F: flat; T: trapezoid; RH: rhomboid horizontal; S: square; RV: rhomboid vertical

**Fig. 3:**
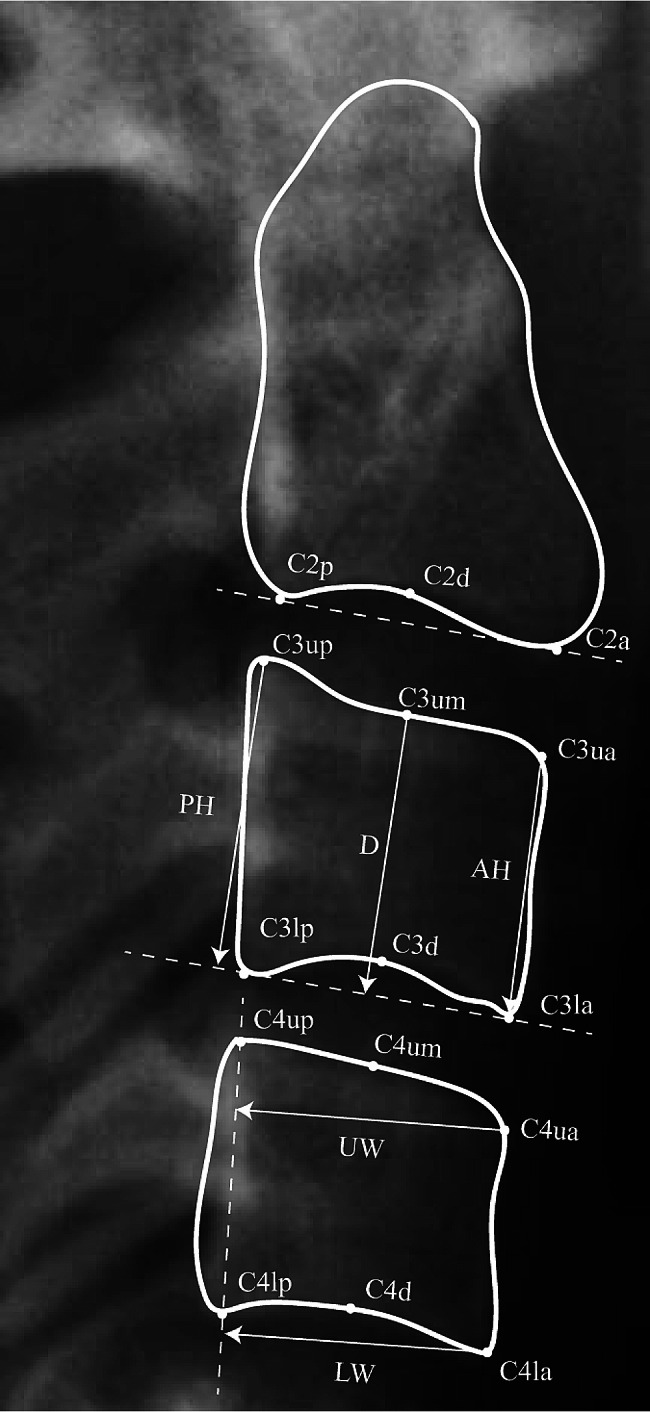
Morphometric assessment of C2, C3, and C4 based on the following landmarks. d, the most superior point of the lower border of the vertebrate’s body; a/la and p/lp, the most anterior and posterior points on the lower border of the vertebrate’s body; ua and up, the most superior points of the anterior and posterior borders of the vertebrate’s body; um, the middle of the upper border of the body. Detailed measuring parameters used in the cephalometric analysis are illustrated in Table 3

A total of 560 films of four CVM stages (iCS1, iCS2, iCS3, iCS4) were obtained, detailed demographic distribution was illustrated in Table 2. The images were randomly apportioned into two separate groups, one for model development (primary cohort, *n* = 480) and the other serving for further testing (validation cohort, *n* = 80). All cephalograms were imported and resized to actual size in ImageJ, with anatomical contours marked on each sample, and the resulting coordinates were then copied into Excel for calculation (Fig. 3). The following parameters were measured for each case: anterior, middle, and posterior vertebral body heights and the anteroposterior body length (Table 3). The anterosuperior angle of C2, C3, and C4 was assessed, as formed with p or lp serving as the vertex. The ratios of these parameters, as defined in Table 3, were calculated.

**Table 2:** Demographic distribution of 560 lateral cephalograms in primary cohort and validation cohort

Characteristic		Primary cohort (*n* = 480)					Validation cohort(*n* = 80)			
Age range	iCS1		iCS2	iCS3	iCS4	iCS1		iCS2	iCS3	iCS4
Min	6.0		7.9	9.9	11.8	7.0		7.2	10.0	12.3
Max	12.2		14.6	17.0	19.0	10.8		13.4	15.4	18.0
Average age	7.6 ± 0.8		10.5 ± 1.1	12.3 ± 1.1	15.7 ± 1.3	7.9 ± 0.4		9.8 ± 1.8	11.3 ± 1.4	15.1 ± 1.8
Number	120		120	120	120	20		20	20	20

**Table 3:** Measuring lines and ratios used in the cephalometric analysis

Parameter	Description
D2	Vertical distance of C2d to the connection of C2a and C2p
D3/4	Vertical distance of C3/4um to the connection of C3/4la and C3/4lp
AH3/4	Vertical distance of C3/4ua to the connection of C3/4la and C3/4lp
PH3/4	Vertical distance of C3/4up to the connection of C3/4la and C3/4lp
UW3/4	Vertical distance of C3/4ua to the connection of C3/4up and C3/4lp
LW3/4	Vertical distance of C3/4la to the connection of C3/4up and C3/4lp
D3/4: AH3/4	Ratio of D3/4 to AH3/4
PH3/4: UW3/4	Ratio of PH3/4 to UW3/4
PH3/4: LW3/4	Ratio of PH3/4 to LW3/4
@2	Antero-superior angle of C2d–C2p connection to C2p–C2a connection
@3–4	Antero-superior angle of C3/4d–C3/4lp connection to C3/4lp–C3/4la connection
C2a-C3ua	Distance between C2a and C3ua
C2p-C3up	Distance between C2p and C3up
C3la-C4ua	Distance between C3la and C4ua
C3lp-C4up	Distance between C3lp and C4up

### Model development

The estimated model based on CVM methods was developed in the primary cohort (*n* = 480). The remaining 80 cephalometric images were used as the validation dataset to further evaluate the performance of the ordinary logistic regression model. As the dependent variable was ordinal in our study, the ordinal logistic regression, underlying strict proportional odds assumption, was utilized to build the primary estimated model. Before selecting CVM features, all features were standardized with Z-score normalization. All variables with statistical significance (*P* < 0.05) selected by univariate analysis were taken as candidates for further logistic regression analyses. The proportional odds assumption was tested using the Brant test, and the variance inflation factor (VIF) was used to measure the severity of multicollinearity in regression analysis. By eliminating non-correlated variables, variables with appropriate correlations were identified, and their functional relationships were extracted to establish the iCS stages.

The constructed logistic regression model yielded individual formulas for each vertebral stage, posing the advantage of determining probabilities (Eq. (1)). P (*y = j|x*) represents the expected probability of each maturational stage in the iCS index (*j = 1,2,3,4*), whereas ${\alpha }_{j}$ denotes the coefficient value as the log-odds, $\beta \left(x\right)$indicates the constant slope coefficient, and *e* is referred to as Euler’s number.

1 $$logit {P}_{j}={\alpha }_{j}+\beta {x}_{j}$$

Equation (2) assisted us in determining the linear function of the independent variables.

2 $$P\left(y=j|x\right)=\frac{1}{1+{e}^{-{\alpha }_{j}+\beta \left(x\right)}}-\frac{1}{1+{e}^{-{\alpha }_{j-1}+\beta \left(x\right)}}$$

### Performance evaluation

Model performance in regard to precision, recall, F1 score, and accuracy was determined based on the primary cohort, supplemented by additional comparative evaluation on the validation dataset. The following index was calculated and recorded based on the confusion matrices: true positive, true negative false positive, and false negative. True positive (TP) refers to correctly classified positive instances, while true negative (TN) denotes correctly classified negative instances. False positive (FP) represents instances that were incorrectly classified as positive, and false negative (FN) refers to instances that were incorrectly classified as negative.

The following metrics were utilized to evaluate the performance of the proposed algorithm:

Precision is the fraction of positive predictions that belong to the positive class.

3 $$Precision=\frac{TP}{TP+FP}$$

Recall is the fraction of positive examples in the dataset that are predicted as positive.

4 $$\text{R}\text{e}\text{c}\text{a}\text{l}\text{l}=\frac{TP}{TP+FN}$$

F1 Score is the harmonic mean of precision and recall.

5 $${\rm{F}}1\,{\rm{score}} = 2 \times \frac{{Precision \times Recall}}{{Precision + Recall}}$$

Accuracy is the fraction of the total correct predictions.

6 $$\text{A}\text{c}\text{c}\text{u}\text{r}\text{a}\text{c}\text{y}=\frac{TP+TN}{TP+TN+FP+FN}$$

### Statistical analysis

All statistical analyses were performed using the IBM SPSS Statistics, v. 23.0 (Armonk, New York), with a *P* value equal to 0.05 as a significant difference. The Shapiro-Wilk test was utilized to check the normality of distribution for quantitative data. Spearman’s correlation analysis was used to assess the correlation between the selected quantitative CVM variables and the CVM stages. A non-parametric test was employed to evaluate the ordinal categorical variables. To identify the significant predictors of the CVM stage, treated as the dependent variable, multivariable ordinal logistic regression was employed. A parallelism test was performed to check the consistency of independent variables in ordinary logistic regression across different CVM stages. The variance inflation factor (VIF) was observed to control the severity of multicollinearity. The confusion matrices that summarize the predicted situation with the actual situation were used to evaluate model performance.

## Results

### Characteristics of the CVM feature

The distribution of the cervical vertebrae maturation stage determined by the researchers’ visual analysis is shown in Table 2. The calculated mean and standard deviation (SD) of each parameter are presented in Table 4. Spearman’s rank correlation coefficient analysis in the primary cohort exhibited 25 parameters significantly correlated by considering the CVM stage as the dependent variable and the measured parameters as the independent variables (*p* < 0.01).

**Table 4:** Descriptive statistics and correlational analysis results of the 25 variables

Parameter	Average (X ± SD)	Correlation coefficient	*P* value
Ages (year)	11.50 ± 3.16	0.939**	<0.01
D2 (mm)	1.17 ± 0.77	0.801**	<0.01
D3 (mm)	1.07 ± 0.80	0.879**	<0.01
AH3 (mm)	8.52 ± 2.76	0.913**	<0.01
PH3 (mm)	10.17 ± 2.24	0.811**	<0.01
UW3 (mm)	11.39 ± 1.35	0.441**	<0.01
LW3 (mm)	12.27 ± 1.38	0.395**	<0.01
D3:AH3	0.11 ± 0.06	0.746**	<0.01
PH3:UW3	0.89 ± 0.17	0.661**	<0.01
PH3:LW3	0.83 ± 0.17	0.681**	<0.01
D4 (mm)	0.86 ± 0.77	0.855**	<0.01
AH4 (mm)	8.29 ± 2.60	0.902**	<0.01
PH4 (mm)	10.22 ± 2.21	0.804**	<0.01
UW4 (mm)	11.56 ± 1.50	0.485**	<0.01
LW4 (mm)	12.33 ± 1.47	0.442**	<0.01
D4:AH4	0.09 ± 0.07	0.779**	<0.01
PH4:UW4	0.88 ± 0.16	0.613**	<0.01
PH4:LW4	0.83 ± 0.16	0.645**	<0.01
@2 (°)	11.41 ± 7.40	0.758**	<0.01
@3 (°)	9.98 ± 7.34	0.852**	<0.01
@4 (°)	7.74 ± 6.89	0.843**	<0.01
C2a-C3ua (mm)	5.47 ± 1.39	-0.626**	<0.01
C2p-C3up (mm)	3.77 ± 1.17	-0.260**	<0.01
C3la-C4ua (mm)	5.54 ± 1.33	-0.670**	<0.01
C3lp-C4up (mm)	3.62 ± 1.08	-0.086**	<0.01

**Significantly correlated parameters based on *P* < 0.01

### Quantitative CVM feature selection and model construction

The primary development of the predicting model was carried out using chronological age and CVM features as input data. As the dependent variable was ordinal in our study, the ordinal logistic regression underlying the strict proportional odds assumption was used in the primary cohort (*n* = 480). The multiple ordinal logistic regression analysis showed that significantly correlated parameters with the cervical vertebral maturational stage were narrowed down to chronological age, D3:AH3, @4, and C3lp-C4up for predicting the CVM stage (*P* < 0.05). The proportional odds assumption has not been violated, and there were no significant multicollinearity issues existed among the selected features in the primary cohort (VIF < 5; tolerance > 2). The coefficient B exhibited in Table 5 represents $\beta$. Followed by calculating each stage probability through the equation series, the estimated *P*_*j*_ value designated the current CVM stage for each individual. The primary model for the prediction of each stage was determined as Eq. (4).

**Table 5:** Multivariate associations determined by ordinal logistic regression

		B		SE (B)	Wald statistic	Sig*	95% CI	
							Lower	Upper
Threshold	[iCS = 1]		32.921	3.452	90.955	0.000	26.156	39.687
	[iCS = 2]		45.460	4.758	91.279	0.000	36.134	54.786
	[iCS = 3]		62.065	6.491	91.430	0.000	49.343	74.787
Location	Ages		3.172	0.336	88.960	0.000	2.513	3.831
	D3:AH3		28.642	5.084	31.743	0.000	18.678	38.606
	@4		0.727	0.094	59.270	0.000	0.542	0.912
	C3lp-C4up		0.453	0.203	4.999	0.025	0.056	0.850

B: Standardized regression coefficient, SE: Standard error, *Significant predictors based on *P* < 0.05

7 $$\begin{array}{l}\beta {x_j} = 3.172 \times {\rm{chronologicalage}} + 28.642 \times \\{\rm{D}}3:{\rm{AH}}3 + 0.727 \times @4 + 0.453 \times {\rm{C}}3{\rm{lp}} - {\rm{C}}4{\rm{up}}\end{array}$$

### Accuracy for stage determination

**Fig. 4:**
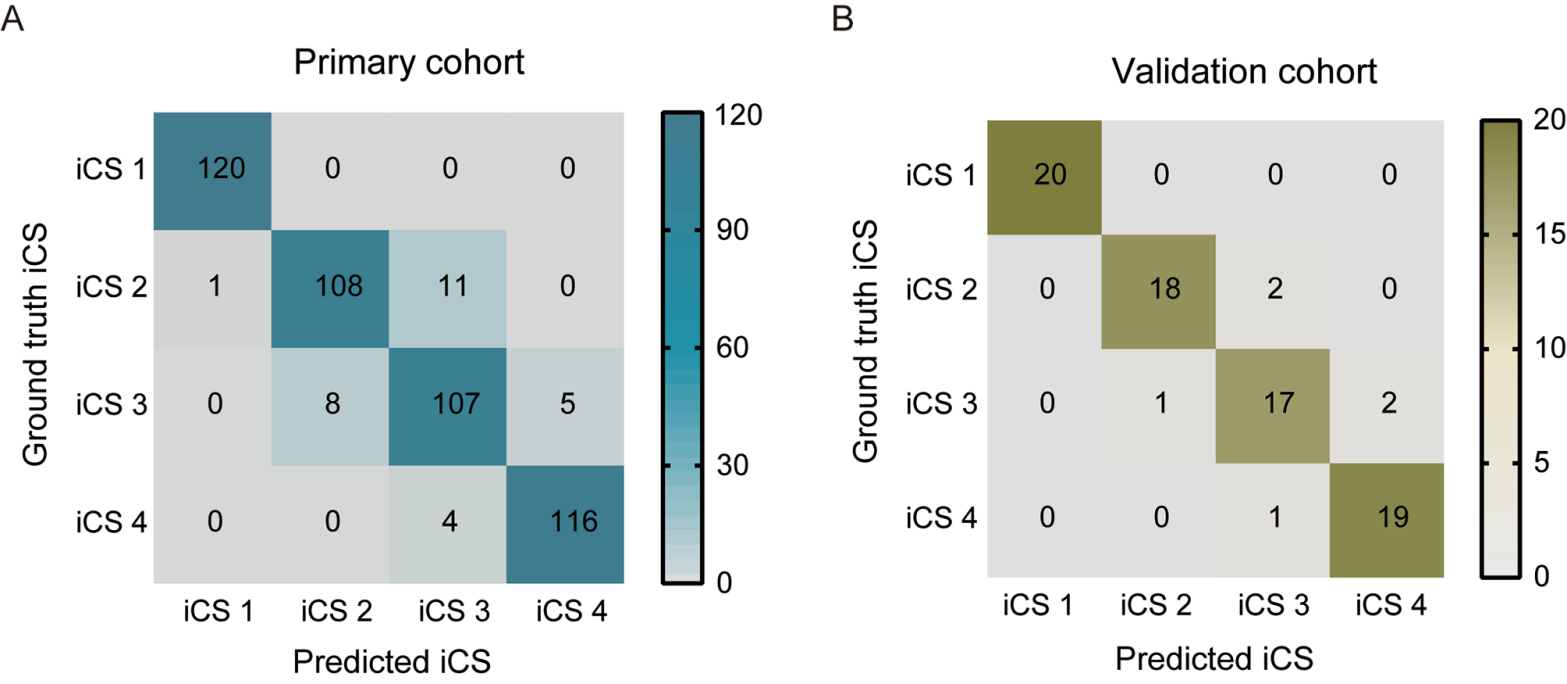
Confusion matrices display the outcomes of primary model performance within the primary cohort (**A**), following fine-tuning on the validation cohort (**B**)

**Fig. 5:**
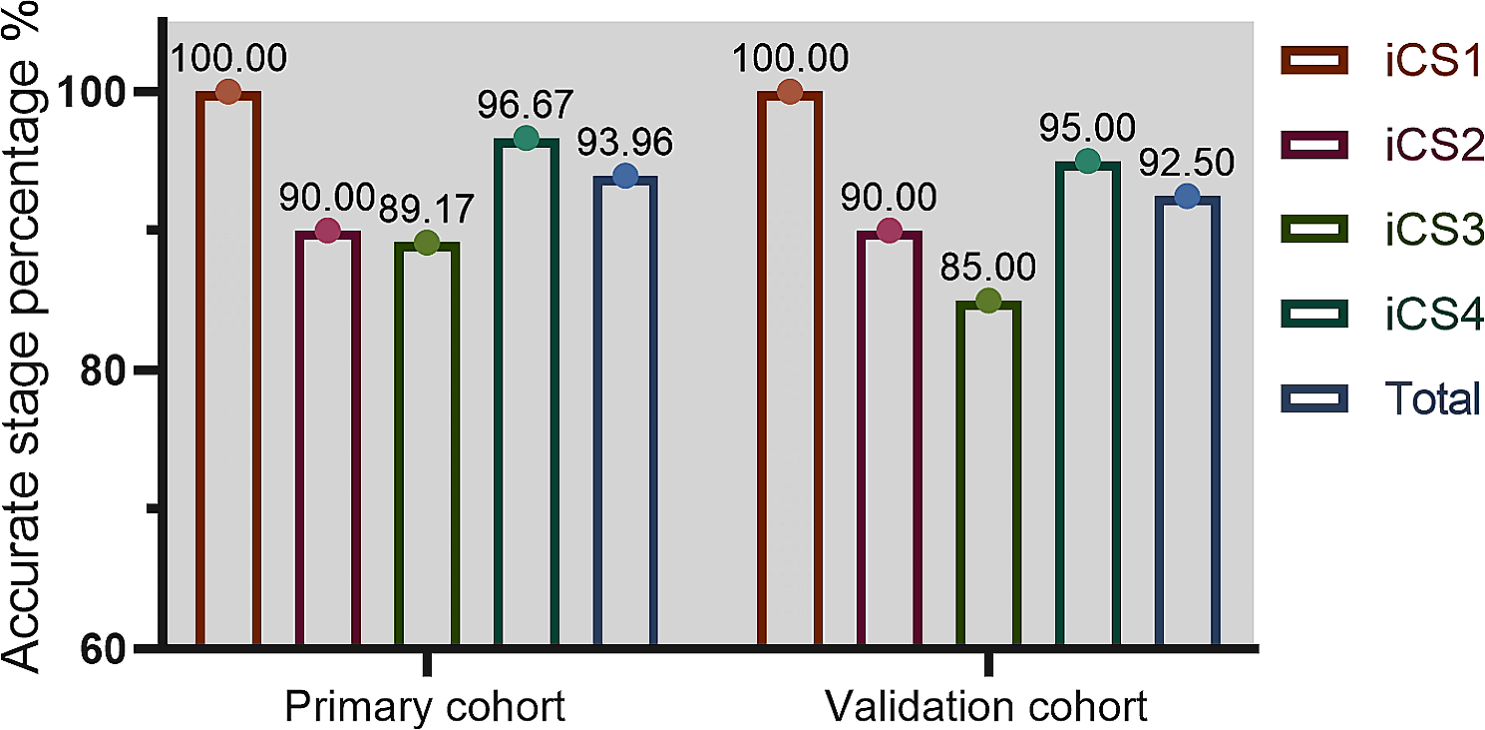
Performance of the primary model using accurate stage estimates

**Fig. 6:**
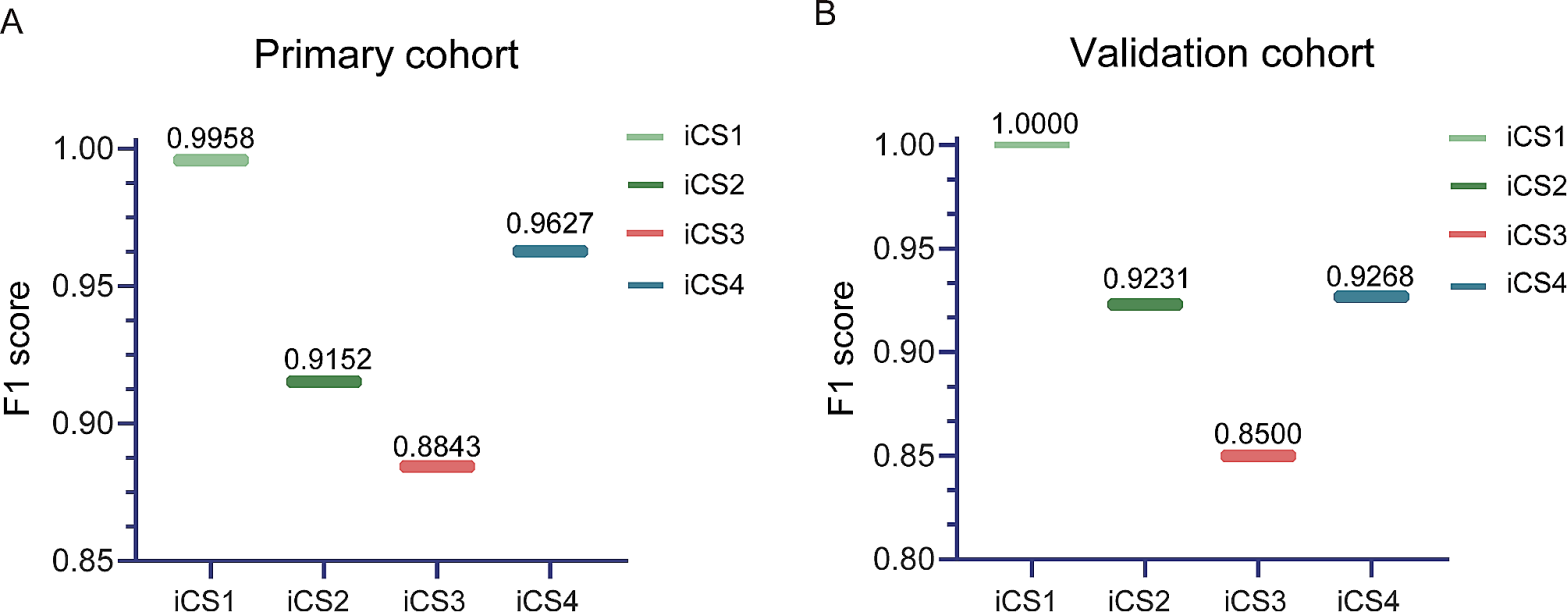
F1 score shows the results of the primary validation and accuracy test in the primary cohort (**A**) and the validation cohort (**B**). The calculated F1 score of each stage was shown above the line

The confusion matrices exhibited a diagonal pattern for prediction, which considers each CVM stage (Fig. 4). The accuracy of the primary cohort in total CVM stage estimating was 93.96%. To validate the diagnostic value of the predictive regression model, we applied a validation data set, and the accuracy was determined to be 92.50%. As displayed in Fig. 5, the initial performance assessment yielded a satisfactory estimation percentage from the corresponding CVM stages in the primary cohort and validation cohort. However, the iCS3 estimate was 89.17% and 85.00%, which failed to meet the estimation threshold we set (90.00%). The F1 score for performance evaluation was determined as Eq. (5), which results in values ranging between 0 and 1, indicative of each stage probability. Comparing the F1 values of the four periods of iCS, we conclude that the discrimination ability of the primary model for each CVM stage is from highest to lowest: iCS1 > iCS4 > iCS2 > iCS3 (Fig. 6; Table 6). Upon thorough inspection, the primary algorithm of CVM mapping attains satisfaction but still needs additional adjustments to improve the performance of iCS3.

**Table 6:** Precision, recall rates, and F1 score of the primary iCS assessment model on the primary and validation cohort for each iCS subgroup

	Primary cohort			Validation cohort		
	Precision	Recall	F1 score	Precision	Recall	F1 score
iCS1	0.9917	1	0.9958	1	1	1
iCS2	0.9310	0.9000	0.9152	0.9474	0.9000	0.9231
iCS3	0.8770	0.8917	0.8843	0.8500	0.8500	0.8500
iCS4	0.9587	0.9667	0.9627	0.9048	0.9500	0.9268

Precision, recall rates, and F1 score are calculated with the confusion matrix in Fig. 4

### Logistic model optimization

Adjusted logistic models were constructed for precisely distinguishing the 3rd stages and improving consistency based on the measurements from 120 subjects in each of the iCS3 and iCS4. As displayed in Table 7, chronological age, @4, and PH4 exhibited statistically significant correlations (*P* < 0.05) and were employed to generate the newly adjusted logistic regression equations based on Eq. (8).

**Table 7:** Multivariate associations determined by modified logistic regression of iCS3 and iCS4

		B	SE (B)	Wald statistic	Sig*	95% CI	
						Lower	Upper
Threshold	[iCS = 3]	51.828	10.143	26.109	0.000	31.948	71.708
Location	Ages	2.139	0.396	29.183	0.000	1.363	2.914
	@4	0.313	0.096	10.657	0.001	0.125	0.501
	PH4	1.444	0.448	10.408	0.001	0.567	2.321

B: Standardized regression coefficient, SE: Standard error, *Significant predictors based on *P* < 0.05

8 $$\begin{array}{l}\beta {x_j} = 2.139 \times {\rm{chronologicalage}} + \\0.313 \times @4 + 1.444 \times {\rm{PH}}4\end{array}$$

**Fig. 7:**
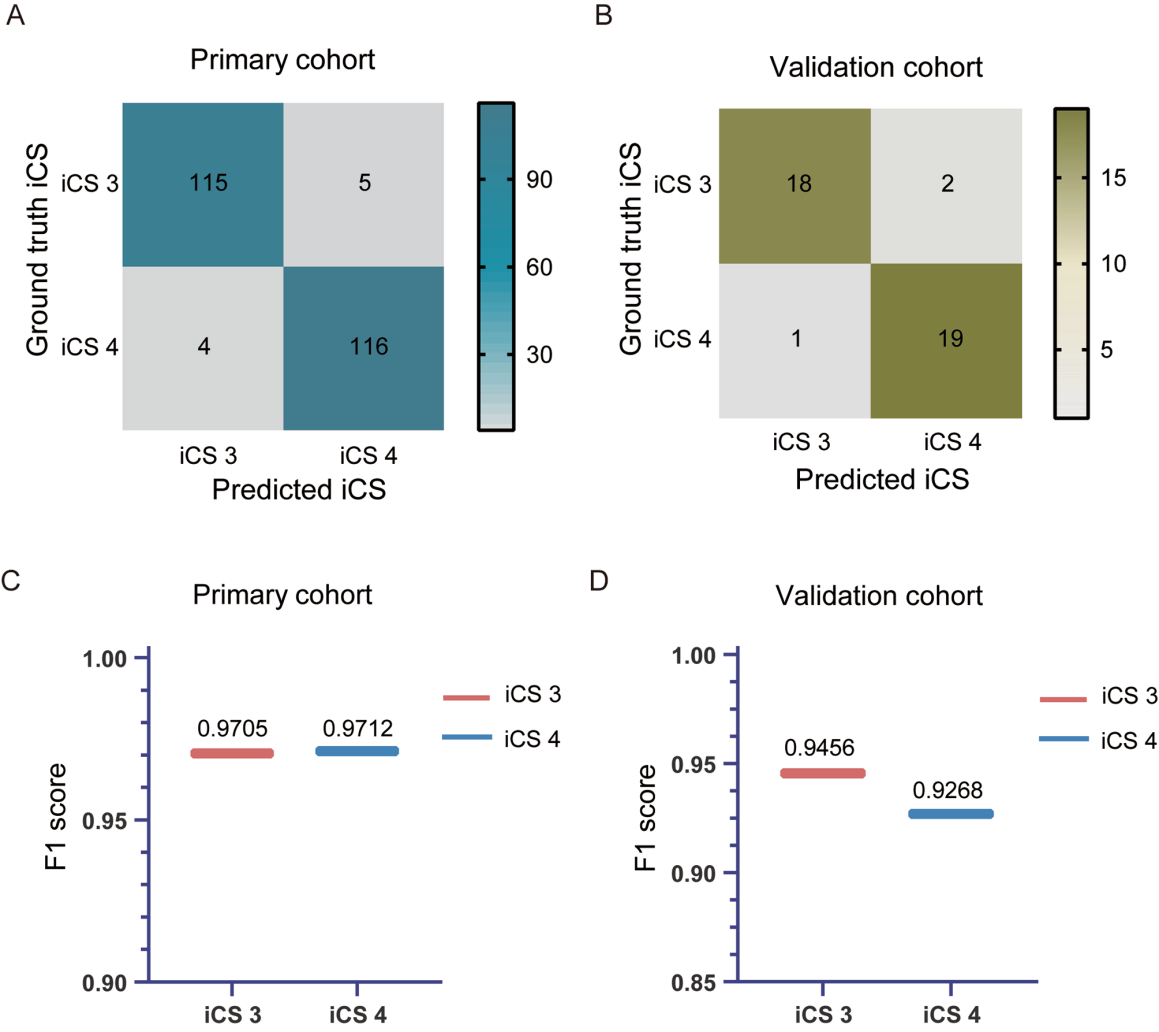
Confusion matrices and F1 score of the optimized model. (**A**) Confusion matrices depict the performance of the optimized model in the primary cohort. (**B**) F1 score corresponding to the optimized model in the primary cohort. The optimized model’s performance is further illustrated in the validation cohort through confusion matrices (**C**) and F1 score (**D**). The calculated F1 score of iCS3 and iCS4 in two datasets was shown above the line

**Fig. 8:**
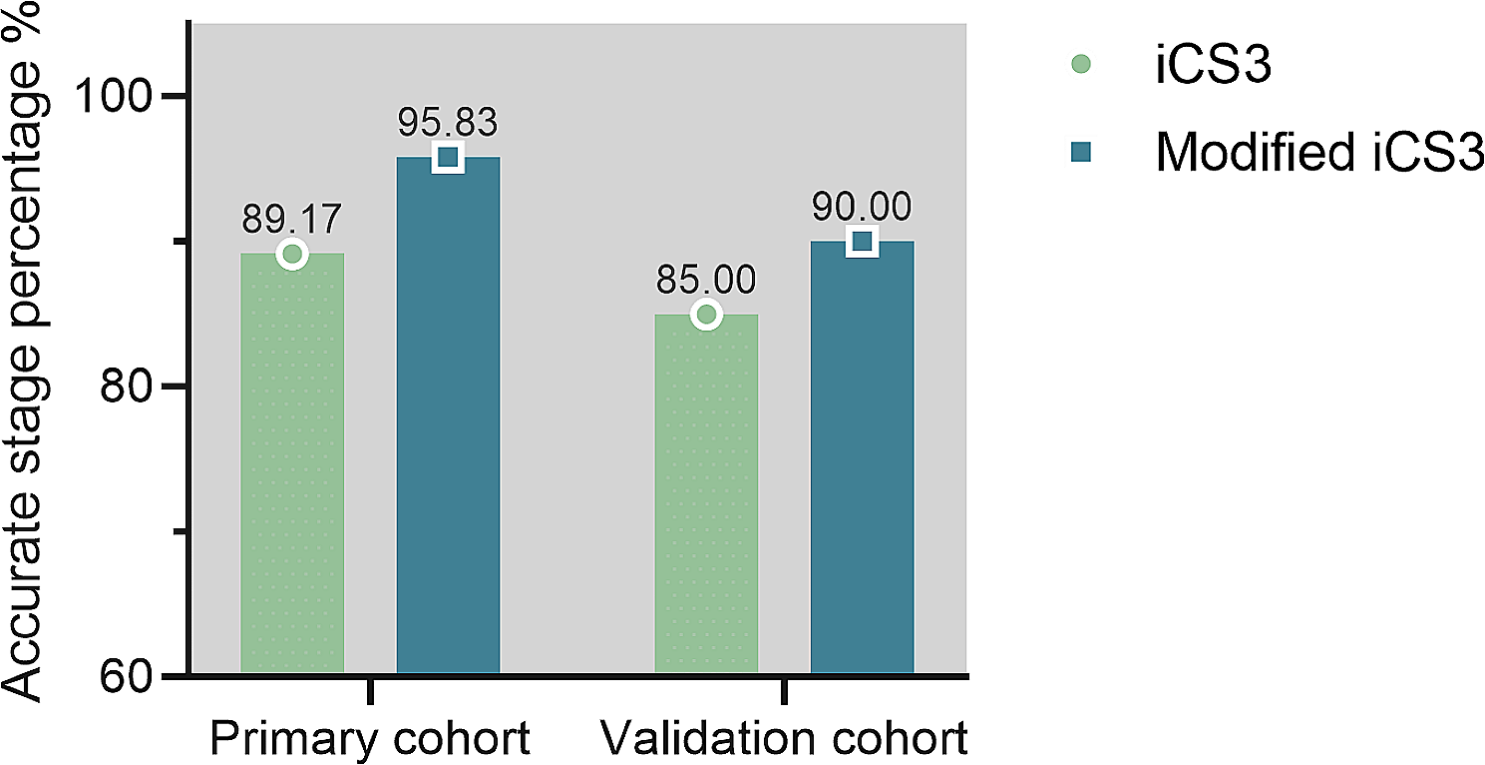
Comparison of model performance of iCS3 in the optimized model of the primary and validation cohort. The estimated stage percentages in the primary and optimized models were displayed above the bar

Subsequently, the accuracy of the finally modified system was obtained using the confusion matrices in two datasets (detailed in Fig. 7A, B, and Table 8). Of the summarized evaluation methods, the optimized algorithm exhibited the overall best performance in the primary and validation cohorts with F1 scores of 0.9705 and 0.9456, respectively (Fig. 7C, D). As illustrated in Fig. 8, an increase in the percentage level of stage estimation accuracy was observed in the performance of adjusted models at iCS3 of the primary cohort (95.83%) and the validation cohort (90.00%), thus indicating the optimized model exhibits a substantial performance enhancement.

**Table 8:** Precision, recall rates, and F1 score of the modified model on the primary and validation cohort for iCS3 and iCS4

	Primary cohort			Validation cohort		
	Precision	Recall	F1 score	Precision	Recall	F1 score
iCS3	0.9829	0.9583	0.9705	0.9474	0.9000	0.9456
iCS4	0.9594	0.9833	0.9712	0.9048	0.9500	0.9268

## Discussion

Evaluation of skeletal maturation is of paramount significance in orthodontic treatment planning and the eventual outcome [22]. Emerging advances in biomathematical modeling on clinical orthodontics motivated us to design multiple ordinal logistic algorithms to objectively score an individual’s cervical stage. In this context, we generated threshold-based ordinal regression models based on anatomic points derived from cephalograms, for detecting and classifying CVM stages, achieving the estimated percentage of 95.83% and 96.64% in two datasets.

The ordinal regression-based method from views of the description degree and the predictive consistency were obtained in our study, thus facilitating the prevalence in orthodontic prognosis. Cervical vertebral growth proceeds from the upper to the lower cervical vertebrae, potentially leading to statistical multicollinearity issues with these parameters. However, our presented ordinary logistic model successfully determined the skeletal maturation level with the absence of multicollinearity problems by accessing the CVM features from C3 and C4 (D3:AH3, @4, and C3lp-C4up). Objective methods of evaluation were constructed using regression formulae based on ratios of measurements in the third and fourth cervical vertebral bodies. In accordance with Santiago et al. work, the logistic model that integrated the parameters of C2, C3, and C4 demonstrated remarkable predictive accuracy albeit a small sample size [23]. However, the challenge lay in accurately assessing the convexity of the iCS3 and iCS4, potentially resulting in low reproducibility [24]. Upon model evaluation, the third stage (iCS3) was moderately difficult to predict accurately, being misinterpreted alongside the adjacent one, prompting us to engage in model optimization. The underlying reason could be that iCS3 is the transition stage from the perpetual peak period to the deceleration period, of which the age range is relatively concentrated and occurs in a short period. Furthermore, the morphological similarities of the bodies of the C3 and C4 and their analogous changeover from rectangular to square form might have projected an indistinctive amount of growth and ultimately confusion in the final model performance in iCS3 estimation, which failed to provide a vertical rectangular shape in the later stages of growth on average [25]. Following that, by further screening and discovering a new significant parameter (PH4) influencing the perpetual stage (iCS3), the accuracy of our proposed model was enhanced to 95.83% and 96.64% in two datasets.

Females are more advanced than males in skeletal maturation. The growth spurt of female adolescents commenced four months earlier and extended for an additional four months compared to males [26]. A disparity between chronological age and skeletal maturity arises, particularly among females, at puberty initiation owing to the secretion of gonadotropin-releasing (GnRH) and sex hormones [27]. In the present study, gender and chronological age were incorporated into the final model. Owing to the existing disparities between females and males during the skeletal maturation, the samples were not grouped by gender, thereby enhancing the robustness of the model [28]. Besides, it has been reported that assessing an individual’s CVM stage with standards from different racial groups may lead to overestimation or underestimation of the degree of maturation. Thus, considering gender and ethnic influences on skeletal maturity, our study exclusively features cephalograms on female adolescents of ethics in the southern and southeast parts of China, which brings new sights into the CVM prediction model.

Recent progress in artificial intelligence (AI) has empowered the execution of tasks such as recognition, segmenting, and classifying in cephalometric radiograph analysis, with the benefit of minimizing interobserver differences [29, 30]. AI-assisted algorithms can perform feature extraction in an automated manner, which allows clinicians to extract confused features with minimal domain knowledge and effort [31]. Kök H et al. trained the artificial neural networks (ANN) model with 300 individuals aged between 8 and 17 years and proposed the ANN algorithm was stable in determining the CVM classes with a 2.17 average rank [32]. With ongoing improvements in AI, Atici SF et al. proposed an innovative deep-learning model with parallel architecture and achieved a validation accuracy of 82.35% in CVM stage classification on female subjects [33]. However, applying deep learning algorithms to medical image analysis presents several unique challenges and obstacles, such as the issues of imbalanced data, deficiency in the confidence interval, and lack of properly labeled data [34, 35]. Due to the complex data structures, AI-aided model training is an extremely expensive endeavor and requires a high computational load, while the medical imaging datasets are too limited when compared to those for general computer vision issues [36]. Besides, deep learning-driven methods failed to mark the actual significant parameters in analyzing films or find the specific drawback area [37, 38]. Another major obstacle to utilizing AI could be the legal ramifications of black-box that physicians build trust and acceptance of something that they may not fully understand, which needs further consideration in clinical application [39]. However, the formula we proposed for each CVM stage simplifies the calculation process for limited indices while maintaining the essence and high accuracy (> 90%) of the estimation, making it suitable for application in clinical practice.

As stated by Houston et al., the use of individual ossification events is of limited use during pubertal growth-spurt prediction [40]. To date, the primary challenge remains in crafting a resilient, versatile, and sensitive diagnostic tool for clinical practice, with a focus on non-invasive methods for assessing skeletal maturity [41]. Established based on significant CVM features, our proposed algorithms could indicate the specific stages directly; thus, the workload of clinicians will be reduced and more objective and consistent evaluation outcomes will be materialized.

The limitation of our study may reside in the patient selection bias stemming from the inherent nature of the retrospective design. However, we tried to intensify the significance and validity of our results by reaching the required sample population during the model development process. The model in our study still warrants further validation and cross-checking, particularly for expanding the testing dataset and objective assessment of skeletal age through artificial intelligence. Additionally, to obtain more reliable outcomes, further longitudinal studies are needed to track individual changes over time.

## Conclusion

The current study provides the following information:

1. Based on the proportional odds assumption, we develop an ordinal regression model for stage determination to achieve an accurate assessment and guarantee the supervision of prediction consistency based on the CVM method.
2. Confusion matrices evaluation of our models demonstrated adequate prediction percentage and commendable recall rate for each stage, thus indicating the appropriate and ideal time for clinical interventions of Chinese female adolescents.

## Data Availability

The datasets used and analyzed during the current study are available from the corresponding author upon reasonable request.

## Abbreviations

AI: Artificial intelligence
ANN: Artificial neural networks
CVM: Cervical vertebral maturation
iCS: Identified cervical vertebral maturation stage
